# Electronic behavioral interventions for headache: a systematic review

**DOI:** 10.1186/s10194-016-0608-y

**Published:** 2016-05-10

**Authors:** Mia Tova Minen, John Torous, Jenelle Raynowska, Allison Piazza, Corita Grudzen, Scott Powers, Richard Lipton, Mary Ann Sevick

**Affiliations:** Department of Neurology, NYU Langone Medical Center, 240 East 38th Street 20th floor, New York, NY 10016 USA; NYU Langone Headache Center, Department of Neurology, NYU School of Medicine, New York, NY USA; Department of Psychiatry, Beth Israel Deaconess Medical Center and Brigham and Women’s Hospital, Harvard Medical School, Boston, MA USA; NYU School of Medicine, New York, USA; Department of Library Services, NYU School of Medicine, New York, USA; Department of Emergency Medicine, NYU Langone Medical Center, NYU School of Medicine, New York, NY USA; Cincinnati Children’s Medical Center, Headache Center, Office for Clinical and Translational Research, Center for Child Behavior and Nutrition Research and Training, Pediatrics Cincinnati, Cincinnati, Ohio USA; Montefiore Headache Center, Department of Neurology, Albert Einstein College of Medicine, Neurology, Bronx, USA; Center for Behavioral Change, Department of Population Health, NYU School of Medicine, New York, NY USA

**Keywords:** Migraine, Headache, Behavioral medicine, Cognitive behavioral therapy, Biofeedback, Progressive muscle relaxation therapy, Electronic

## Abstract

**Background:**

There is increasing interest in using electronic behavioral interventions as well as mobile technologies such as smartphones for improving the care of chronic disabling diseases such as migraines. However, less is known about the current clinical evidence for the feasibility and effectiveness of such behavioral interventions.

**Objective:**

To review the published literature of behavioral interventions for primary headache disorders delivered by electronic means suitable for use outside of the clinician’s office.

**Methods:**

An electronic database search of PubMed, PsycINFO, and Embase was conducted through December 11, 2015. All eligible studies were systematically reviewed to examine the modality in which treatment was delivered (computer, smartphone, watch and other), types of behavioral intervention delivered (cognitive behavioral therapy [CBT], biofeedback, relaxation, other), the headache type being treated, duration of treatment, adherence, and outcomes obtained by the trials to examine the overall feasibility of electronic behavioral interventions for headache.

**Results:**

Our search produced 291 results from which 23 eligible articles were identified. Fourteen studies used the internet via the computer, 2 used Personal Digital Assistants, 2 used CD ROM and 5 used other types of devices. None used smartphones or wearable devices. Four were pilot studies (*N* ≤ 10) which assessed feasibility. For the behavioral intervention, CBT was used in 11 (48 %) of the studies, relaxation was used in 8 (35 %) of the studies, and biofeedback was used in 5 (22 %) of the studies. The majority of studies (14/23, 61 %) used more than one type of behavioral modality. The duration of therapy ranged from 4–8 weeks for CBT with a mean of 5.9 weeks. The duration of other behavioral interventions ranged from 4 days to 60 months. Outcomes measured varied widely across the individual studies.

**Conclusions:**

Despite the move toward individualized medicine and mHealth, the current literature shows that most studies using electronic behavioral intervention for the treatment of headache did not use mobile devices. The studies examining mobile devices showed that the behavioral interventions that employed them were acceptable to patients. Data are limited on the dose required, long term efficacy, and issues related to the security and privacy of this health data.

This study was registered at the PROSPERO International Prospective Register of Systematic Reviews (CRD42015032284) (Prospero, 2015).

## Introduction

Experts in behavioral headache medicine have identified ten areas of critical need for behavioral headache research. One important unmet need is the development of an innovative technology-based treatment platform for headache self-management [1]. Behavioral headache treatments (e.g., progressive muscle relaxation (PMR), biofeedback, and cognitive-behavioral therapy (CBT)) are Level A Evidence-Based migraine treatments [2] that are essentially free of side effects [3]. Behavioral treatments have enduring benefits [4] and may be less costly than pharmacologic interventions [5]. Questions remain whether these evidence-based treatments can effectively be delivered electronically so that patients can do them on their own outside of the clinical setting. This is an important and timely topic because in 2014, 64 million Americans had smartphones [6]. The FDA states that per industry estimates, 500 million smartphone users worldwide will be using a health care application (app) by 2015 [7], and by 2018, 50 percent of the more than 3.4 billion smartphone and tablet users will have downloaded mobile health apps [8]. Most published literature regarding health apps has focused on preventing and managing chronic disease [9, 10], monitoring app acceptability and utility [11–14], and qualitative studies of user experience and desired functions [15–18]. However, it is unclear the extent to which apps are effective at facilitating behavior change [19].

The creation of electronic headache apps has proliferated, with over 40 headache apps in the U.S. Google Play Store (Date accessed 12/23/15) and over 70 headache apps in the U.S. Apple iTunes store (Date accessed 1/8/16). These apps advertise various purposes-the ability to track headache frequency with electronic headache diary functionality, the ability to detect headache triggers, and the ability to treat headaches with behavioral treatments. Prior studies have demonstrated that smartphone apps with electronic headache diaries are a reliable method for data collection preferred over paper headache diaries by patients because electronic diaries are more discreet in the work place [20]. Electronic headache diary data collection also results in fewer secondary data errors [21], less administrative burden [22, 23], high participant acceptance [23], and potential cost savings [24]. Additionally, this format allows the use of reminders and timely follow-up of non-compliant participants via real time investigator data monitoring capabilities. Thus, while we know that electronic headache diaries are useful for collecting data, it is unknown whether behavioral interventions, oftentimes delivered along with the electronic diaries, are feasible and effective.

The purpose of this systematic review is to describe the current body of literature on electronic behavioral interventions for primary headaches that can be used outside of the clinician’s office. Specifically, we reviewed the literature to first examine the modalities in which such treatments were delivered (computer, smartphone, watch and other), types of behavioral intervention delivered (CBT, biofeedback, relaxation, other), the headache type being treated, duration of the treatment, adherence, and outcomes obtained by the trials. We also examine the overall feasibility of smartphone interventions for primary headache disorders. Understanding the existing literature on the electronic behavioral interventions will increase our understanding of what has been learnt so far, where the potential of this technology may be best realized in future, and what may be applied to the growing field of mHealth.

## Review

### Methods

A health sciences librarian (AP) conducted searches in PubMed.gov, Embase (via Ovid), and PsycINFO (via Ovid) from January 1, 2000 through December 11, 2015. The search strategy combined three concepts: (1) headaches (e.g. “migraines”), (2) electronic or computerized formats (e.g. “internet”), and (3) behavioral interventions (e.g. “behavior therapy”). Terms were searched both as keywords and Subject Heading terms. The search was restricted to studies published in or after 2000. A manual search among references of selected articles and reviews was also performed (MM). The full search strategies are available in the Appendix. After deduplication, 291 unique records were identified.

Studies were considered eligible for inclusion if they were randomized controlled trials, prospective non-randomized trials, or observational studies using a behavioral intervention for a primary headache disorder. Secondary headache disorders, including dental disorders, were not eligible. Studies involving telephonic interventions were not included.

Two study investigators (MM, JT) included or excluded articles based on the predefined eligibility criteria using a two-step procedure. In the first step, the investigators independently reviewed titles and abstracts. Twenty-two articles were resolved by consensus, and 256 articles were excluded. Main reasons for exclusion based on title and abstract were because the papers did not pertain to a primary headache disorder (*N* = 99), were review articles (*N* = 78), or were studies about medications (not behavioral interventions) (*N* = 25).

In the second step, the same investigators independently reviewed the full-text version of the remaining 32 eligible articles. Any differences in selection from the two independent searches were resolved by consensus between the two investigators. Of the 32 remaining articles for full-text screening, 23 met inclusion criteria. Two articles were resolved by consensus.

A standardized form was used for data extraction, including the following items: first author, year of the study, country where the study was performed, type of behavioral intervention, mode of electronic delivery, study design, recruitment and setting, headache type, participants, outcomes measured, and results.

**Fig. 1:**
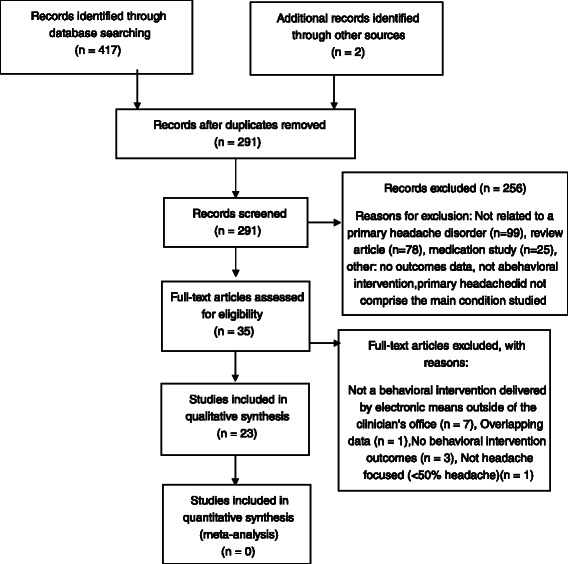
Preferred reporting items for systematic reviews and meta-analyses (PRISMA) flow diagram

This study was registered at the PROSPERO International Prospective Register of Systematic Reviews (CRD42015032284) [25]. Reporting of this study conforms to the Preferred Reporting Items for Systematic Reviews and Meta-Analyses (PRISMA) statement [26] and the flow diagram can be found in Fig. 1.

### Results

As indicated in Table 1, 23 studies met our search criteria, 8 were performed in pediatric populations and the remainder performed in adults. The majority (14/23, 61 %) used the internet to deliver the behavioral electronic intervention. Only 2/23 (9 %) used CD ROMs and 2/23 (9 %) used Personal Digital Assistants (PDAs). A PDA is a handheld digital device which is similar to its successor of the smartphone but often lacks either the touch screen, connectivity, or ability to download and run native applications that a smartphone can. Five used alternate means but none used smartphone technology. About half (13/23, 57 %) were randomized controlled trials. For the behavioral intervention, CBT was used in 11/23 (48 %) (one study used family CBT), relaxation was used in 8/23 (35 %) (half of these studies specified that PMR was used for the relaxation therapy), and biofeedback was used in 5/23 (22 %) of the studies. The majority (14/23, 61 %) used more than one type of behavioral modality. Descriptions of the procedures for teaching and/or practicing the CBT were varied. Descriptions ranged from “a CD ROM program designed to give children additional strategies to help manage their headache pain” to references of full CBT procedure modules. The table in the Appendix illustrates the varied descriptions provided in the papers. The duration of therapy ranged from 4–8 weeks for CBT with a mean of 5.9 weeks. The duration of other behavioral interventions ranged from 4 days to 60 months.

**Table 1:** Description of studies meeting eligibility for the systematic review

Tool	Type of behavioral intervention	Design	First author, year	# of participants (N), setting, duration of treatment	Headache type/criteria	Outcomes measured	Results (HA freq, HA intensity, disability, adherence)	Other results	Drop out rate
CD ROM	CBT	RCT (waitlist control)	Connelly, 2005 [44]	*N* = 50 (ages 7–12) Pediatric Neurology Clinic 4 weeks	Migraine, tension type or chronic daily HA min 4/month with symptom free period, assessed by neurologist or NP	HA duration; HA days; HA intensity; HA severity; Medication; Self-efficacy; QoL; Disability; Acceptability	• There was a significant reduction in HA frequency from baseline to post-tx in both the tx (Headstrong) + control groups (Univariate ANOVA, *p* < 0.001) with a trend suggesting a greater reduction in the tx group (60.16 % reduction in tx group vs. 45.43 % reduction in controls, *p* = 0.091).	• Significant “group by phase” interaction effect on the HA duration variable (*p* = 0.014) suggesting that there was different changes in HA duration from baseline to 1-month post-tx as a function of group assignment.	6 % Overall (4 % tx group vs 2 % control group)
								• A clinically significant change in HI from baseline to 1 month post-tx was observed in 60 % of the tx group + only 8 % of the control, therefore using the adjunctive Headstrong program resulted in more children achieving clinically significant outcomes (Chi-square, *p* = 0.005)	
							• Significant “group by phase” interaction effect on the HA intensity variable (*p* = 0.004) suggesting that there was different changes in HA intensity from baseline to 1-month post-tx as a function of group assignment.		
							HA intensity decreased from baseline to 1 month post-tx in the tx group, while it remained fairly constant in the control group.		
							• No power to assess secondary outcomes including disability.		
	CBT + self-management	RCT	Rapoff, 2014 [45]	*N* = 35 (ages 7–12) Pediatric clinics & children’s hospitals 4 weeks	migraine with or without aura min 1/week	HA duration; HA frequency; HA days; HA severity; QoL; Disability	• NS change in HA frequency between tx + control groups		50 % Overall-no allocated intervention (55 % tx group vs. 43.3 % control group)
							• There was a statistically significant difference in pain severity (10-point VAS) post-intervention, with tx group reporting lower pain severity than control group (5.06 vs. 6.25, *p* = 0.03, ES = 0.7).		
									18.6 % Overall were lost to follow-up (17.5 % tx group vs. 20 % control group)
							• At 3 months post-intervention, parents reported lower migraine-related disability (PedMIDAS) in the tx group compared to control group (1.36 vs. 5.18, *p* = 0.04).		
Internet	CBT	Parallel group unblinded RCT	Day, 2014 [46]	*N* = 36 (ages 19+) Physician referral, brochures + public service announcements 7 weeks	Migraine, Tension-type, cluster or other primary HA min 3 days/month	HA duration; HA index; HA frequency; HA intensity; HA severity; Medication; Self-efficacy; Disability; Acceptability; Alliance; Feasibility; Engagement; Other	• There was a statistically significant baseline to post-test decrease in HA frequency, HA peak intensity + HA average intensity in the total completer sample, however there were no significant differences in these variables between the tx + control groups.	• ITT analysis: Greater improvement in self-efficacy (*p* = 0.02) + pain acceptance (*p* = 0.02) in tx group compared to controls.	11.3 % Overall prior to randomization
									2.1 % Overall after randomization (.53 % in tx group vs. 1.6 % in control group)
								• Completer analysis: Improved pain interference (*p* < 0.01) + pain catastrophizing (*p* = 0.03) in tx group compared to controls.	
							• For the ITT analysis, there was a significant decrease in HA frequency overall, but again no difference in tx groups.	• MBCT was found to be feasible, tolerable + acceptable to patients.	
	CBT	Prospective parallel group design	Bromberg, 2012 [27]	*N* = 213 (ages 18–65) Website postings, electronic newsletter announcements, neurology clinics, + social networking/community sites 4 weeks	migraine with or without aura min 2/month	Pain catastrophizing; Self-efficacy; Disability; LoC; CPC; DAS; Other	• Reduction of HA frequency + severity could not be tested due to technical problems resulting in loss of data.	• Decrease in depression (DASS) in tx group compared to controls from baseline to 3-months post-intervention (*p* = 0.0009) + baseline to 6-months post-intervention (*p* = 0.0079).	11.3 % Overall prior to randomization
									2.1 % Overall after randomization (.53 % in tx group vs. 1.6 % in control group)
							• Both tx + control subjects reported similar reductions in disability on MIDAS (12.8 % decrease + 13.0 % decrease respectively) immediately post-intervention.		
								• Decrease in stress (DASS) in tx group compared to controls from baseline to post-intervention (*p* = 0.0324) + from baseline to 3 month follow-up (*p* = 0.0045).	
							• Follow-up assessment completion in tx vs. control groups respectively were 80 % vs. 89 % at 1-month, 70 % vs. 82 % at 3-months, + 55 % vs. 82 % at 6-months.	• Reduction in pain catastrophizing (PCS) in tx group compared to controls from baseline to post intervention (*p* = 0.0030), 3-month follow up (*p* = 0.0099), + 6-month follow up (*p* = 0.0006).	
								• CPCI-42:Increase in relaxation (baseline to post-intervention, 3-month assessment + 6 month assessment), task persistence (baseline to post-intervention + 3-month assessment), exercising (baseline to post-intervention) + use of social support (baseline to post-intervention) in tx group compared to controls.	
								• Increases in self-efficacy in tx group compared to controls (baseline to post-intervention, 3-month assessment + 6 month assessment)	
	CBT (family-based)	RCT	Law, 2015 [43]	*N* = 83 (ages 11–17) Pediatric clinic 8 weeks	Recurrent HA (>3 months)	HA days; HA intensity; Activity limitation; DAS; Acceptability; Feasibility; Engagement; Other	• There was a statistically significant reduction in HA frequency from baseline to post-tx + baseline to 3-month follow up in both tx conditions, however there was NS difference in HA frequency between tx + control groups.	• There was a significant reduction in activity limitations, emotional functioning + parent response to pain behavior from baseline to post tx in both groups, but no significant difference between tx + control groups	28.9 % Overall (29.5 % in tx group vs 28.2 % in control group
							• There was a statistically significant reduction in HA pain intensity from baseline to post-tx + baseline to 3-month follow up in both tx conditions, however NS in HA frequency between tx + control groups.		
	CBT + PMR	RCT	Trautmann, 2010 [47]	*N* = 65 (ages 10–18) Newspaper ads, websites 6 weeks	Migraine, tension-type HA, or combined HA min 2 HA attacks/month	HA duration; HA frequency; HA intensity; Pain catastrophizing; QoL; DAS; Acceptability; Alliance; Other	• There was a significant reduction in HA frequency + duration post-tx in all groups, but NS between groups.	• Pain catastrophizing was significantly reduced post-assessment in all groups, but no difference was found between groups.	7.7 % Overall (16.6 % in CBT group vs. 0 % in AR group vs. 5.3 % in EDU group)
							• No significant difference in HA intensity was found in any group at post-assessment	• Responder rates (reduction in HA frequency of 50 % or more from baseline) were significantly higher in CBT (63 %) + AR (32 %) groups, compared to the EDU/ control group (19 %).This resulted in NNTs of 2.0 for CBT + 5.2 for AR.	
							• There was no significant difference in depression, psychopathological symptoms, + health-related quality of life in any group post-assessment.		
	CBT + Relaxation	RCT	Sorbi, 2015 [48]	*N* = 368 (18–65) HA centers, website, + flyers 8 weeks*	Migraine with 2–6 attacks in the month prior to randomization	HA index; HA intensity; Medication; Self-efficacy; QoL; Disability; LoC; Other	• NS in HA frequency or intensity in either group or between groups.	• HA duration decreased significantly more in telephone arm (*p* < 0.05)	32 % Overall (29 % tx vs. 35 % control)
								• NS in HI between groups	
								• Self-reported inventories (HADS depression subscale, HDI, PSS) showed significant improvements in both groups but not between groups.	
	Multimodal including CBT	RCT	Trautmann, 2008 [49]	*N* = 18 (ages 10–18) Participation was online, recruitment strategy not specified 6 weeks	Migraine +/or tension-type HA min 2 HA attacks/ month	HA duration; HA frequency; HA intensity; Pain catastrophizing; Acceptability; Alliance	• No significant difference found between HA frequency or intensity between groups post-tx.	• NS found between the two groups post tx in any of the outcome variables (HA frequency, intensity, duration, or pain catastrophizing).	11.1 % Overall (5.6 % in tx vs. 5.6 % in control)
							• Frequency of HA decreased significantly from pre-tx to post-tx in CBT group but not in control (EDU) group.	• Pain catastophizing was significantly decreased from baseline to post tx in CBT group but not in control.	
								• NS difference between the groups in satisfaction or “patient-therapist-alliance/assistance”	
	Multimodal including CBT	RCT	Hedborg, 2012 [50]	*N* = 76 (ages 22–65) Newspaper ads 24 weeks (MBT) + 36 weeks (hand massage)	Migraine at least 2 times monthly	Medication		• Decrease in total migraine drug intake at the end of the MBT program in the MBT group (13.0 vs. 10.1 drug doses/subject/56 days) compared to controls, no significant difference in total migraine medication drug intake in the control group (8.3 vs. 8.9 drug doses/subject/56 days)	8.4 % Overall (7.4 % in MBT+ hand massage vs. 14.3 % in MBT vs. 3.6 % in control group)
								• Drug efficacy increased during MBT from 0.30 to 0.52 (*p* < 0.001), but this was mainly explained by the increase proportion of mild HAs	
	Multimodal including CBT	RCT	Hedborg, 2011 [51]	*N* = 83 (ages 22–65) Newspaper ads 24 weeks (MBT) + 36 weeks (hand massage)	Migraine at least 2 times monthly	HA frequency; QoL; DAS; Acceptability		• 40 % of patients receiving MBT alone + 42 % of patients receiving MBT+ hand massage had 50 % + reduction in migraine frequency when compared to control group.	8.4 % Overall (7.4 % in MBT+ hand massage vs. 14.3 % in MBT vs. 3.6 % in control group)
								• Hand massage NS on migraine frequency compared to MBT alone.	
								• NS in depression (MADRS-S) scores from baseline to post-tx or across groups.	
								• Improvement in “perceived work performance” in hand massage + MBT group from baseline to all follow-up points.	
	Multimodal including CBT + applied relaxation	RCT	Andersson, 2003 [38]	*N* = 44 (ages 18–59) Newspaper ads, project website 6 weeks	Migraine, Tension-type HA, or cluster HA (Dx = self-report)	HA duration; HA index; HA days; HA intensity; Disability; CPC; DAS	• NS in HA frequency or intensity (either group or between groups).	• HA duration decreased more in telephone arm (*p* < 0.05)	32 % Overall (29 % tx vs. 35 % control)
								• NS in HI between groups	
								• Self-reported inventories (HADS depression subscale, HDI, PSS) showed significant improvements in both groups but not between groups.	
	Multimodal including PMR + biofeedback	RCT (delayed tx control which later crossed over)	Devineni, 2005 [52]	*N* = 139 (age not specified) Internet-based promotion channels e.g. online classified ads, websites 4 weeks	Migraine with or without aura, tension-type HA, or mixed.	HA duration; HA index; HA frequency; HA severity; Medication index; Disability; DAS; Cost	• Only a non-significant trend was found for # of HA days per week between groups post-tx.	• There was a trend towards a between group difference in medication index post tx (*p* = 0.12)	38.1 % Overall (58.8 % in immediate tx group vs. 70.4 % in delayed tx group)
								• % of tx completers with clinically significant improvement (50 % decrease in HI) was 38.5 % vs. 6.4 % (waitlist)	
							• There was a significant decrease in peak intensity between the tx and control groups post-tx		
								• Estimated time expenditure for the therapist =1.3 h/participant (range = 0.2–8.8 h), resulting in a cost-effectiveness estimate of 0.32.	
								• Greater compliance was associated with greater improvement in primary HA outcomes.	
	Multimodal including PMR	Randomized to intervention vs waitlist	Strom, 2000 [53]	*N* = 102 (ages 19–62) Newspaper articles + Internet magazines 6 weeks	Recurrent HA (>6 months, at least 1 HA per week)	HA duration; HA index; HA days; HA intensity; HA severity; Medication index; Disability; DAS; Cost	• Decrease in HA days + HA peak intensity post-tx in the tx group compared to the control group.	• Improvement in HI (average reduction in HI was 31 % for tx group vs. 3 % for control group, *p* = 0.028).	56 % Overall
								• Cost-effectiveness: Estimated sum of therapist time = 40 hrs/participant. Cost-efficiency estimate: 0.78	
							• NS in Headache Disability Inventory (HDI) or Beck Depression Inventory (BDI).		
	Multimodal including relaxation	RCT	Kleiboer, 2014 [54]	*N* = 368 (ages 18–65) HA specialist referral, website, flyers, newspaper/ magazine ads 8 weeks-11.4 weeks, 8 lessons, to be done in 7–10 days	Migraine with or without aura + 2–6 attacks/30 days prior to randomization	HA frequency; HA days; HA severity; Self-efficacy; QoL; Disability; LoC	• A 20–25 % decrease in migraine frequency was found for both the tx + control groups, NS between groups.	• BT (tx group) had significantly more improvement that the control group in migraine-related self-efficacy (*p* < 0.001, ES = 0.86), + developed more internal control (p,0.001, ES = 0.57) but less external control (*p* < 0.001, ES = 0.78).	27.4 % Overall (39.0 % in tx group vs. 14.5 % in control group)
							• A significant but small decrease in average attack peak intensity was seen in the ITT BT (tx) group from baseline to post-tx, but NS between groups.		
							• Compliance was explored in a random sample of 60 participants, which showed that participants reported conducting at least one relaxation exercise on 45.5 % of days of being in training.		
	Other	Descriptive study	Sorbi, 2010 [55]	*N* = 10 (ages 31–68) Individuals with recently expressed interest in self-management training 10 weeks	Migraine with 1–6 attacks/month	Acceptability		• All lessons were rated positively regarding clarity, instructiveness, importance + easy execution by new participants	40 % Overall (New Participants) + 0 % overall (Expert Patients)
								• Expert patients provided positive ratings for the web application, digital support, + web-adaptation of the protocol.	
	Self-Management Program	Descriptive study (Interviews + concept mapping to develop Web-prototype + study feasibility)	Donovan, 2013 [56]	*N* = 12 (ages 12–17, adolescents), 9 (ages 30–55, caregivers) 12 (adults, clinicians).** Newspaper ads + community message board (adolescents + caregivers). Emails/invitation at conference (clinicians) 60 min (interview) + 30 min (acceptance testing via telephone)	Migraine	Acceptability		• Disagreement over content areas for the website-clinicians but not adolescents felt diet + exercise were important to include.	N/A
								• During the prototype evaluation, most adolescents indicated that the website would be useful (especially the “Ask an Expert” feature) when they felt a migraine coming on or had a migraine.	
								• Caregivers reported being “somewhat” to ‘extremely likely” to use the range of features offered on the website.	
PDA	Multimodal including relaxation	Descriptive Study + Case Control study	Kleiboer, 2009 [57]	*N* = 44 (ages 25–63)*** HA websites, newspaper ad, referral by HA specialists 3 weeks	Migraine	HA frequency; QoL; LoC; Acceptability	• There were no significant improvements in HA frequency in the ODA + BT group (tx) compared to BT alone (control).	• ODA was considered feasible, well-accepted + perceived to support self-care.	29.5 % Overall
								• There were no significant improvements in internal control or migraine-specific QoL in the ODA + BT group compared to BT alone.	
	Other	Descriptive pilot study (To establish feasibility)	Sorbi, 2007 [39]	*N* = 5 (ages 24–52) Unknown 8.5 days on average (range 4–12 days)	Migraine without aura	Acceptability	• In the second run, adherence was 85 %.	• ODA had good acceptability evidenced by positive participant responses	0 % Overall
								• Loss of data due to technical problems amounted to 6.8 % of potential diary entries + lost internet connection contributed to loss of 5.6 % of lost diary entries.	
Other	Multimodal including biofeedback	Prospective, single-arm, open-label pilot study	Shiri, 2013 [58]	*N* = 10 (ages 10–17.5) Pediatric neurology clinic 6 months- 60 months	Chronic migraine or Chronic Tension-type HA	QoL; Activity limitation; Other	• Patients reported a decline in HA severity (VAS 4.28 pre-test vs. 3.11 post-test, p 0.015) + signficant improvements in daily function + quality of life.	• Improvement pre-tx to post-tx in quality of life (Pedsi QL) + daily function (measured by 2 questions on VAS scale)	10 % Overall
								• Overall the participants reported they were satisfied with the tx.	
	Multimodal including Biofeedback	RCT	Scharff, 2002 [59]	*N* = 36 (ages 7–17) Referred from children’s hospital 6 weeks	Migraine with or without aura +/-co-existing tension-type HA	HA index; HA days; HA severity; DAS; Acceptability	• Change in # of HAs recorded + highest intensity rating over time, but there were no significant between-group differences. Likely due to small n + low power of the study.	• 53.8 % (7) of children in the handwarming biofeedback group, 10 % (1) in the handcooling biofeedback group, and 0 %(0) in the waitlist control group had a 50 % or more decrease in HI at the post tx. The significantly higher proportion of participants achieving 50 % reduction in HI in handwarming group vs. handcooling group was maintained at 3 month + 6 month follow-up.	9.4 % Overall (0 % in handwarming group vs. 9.1 % in handcooling group vs. 8.3 % in WLC group)
							• Adherence: Data from home practice records of 29 participants in handwarming or handcooling group indicated the average # of practice sessions was 5.3 times per week.		
								• NS in CDI or STAIC scores.	
								• There was a temperature change between the handwarming + handcooling groups, with the handwarming group more likely to report that their temperatures increased.	
	Other-Sound therapy	RCT (double blind, placebo-controlled study with a parallel group add-on design)	Trinka, 2002 [60]	*N* = 32 (ages 16–60) Outpatient HA clinic 12 weeks	Migraine with or without aura assessed by neurologists	DAS; Acceptability; Other		• Raw values of the “headache” subtest of the GBB improved in both groups but NS between groups.	No Adherence Data
								• NS in FPI-R, STAI or SDS	
	PMR+ Biofeedback	Prospective non randomized	Arena, 2004 [61]	*N* = 4 (ages 52–64) Medical Center 8 weeks	Migraine or combined migraine-tension HA	HA index; HA days; HA severity; Medication index	• 1 subj had 50 % or greater reduction in HI, 2 had some clinical improvement, 1 subject demonstrated no tx response		0 % Overall
	Biofeedback	Prospective non randomized	Folen, 2001 [62]	*N* = unknown**** U.S Army/Navy hospitals Not specified	Migraine, Chronic daily HAs	Acceptability		• When evaluating the viability of the system in 2 separate rooms of the medical center, patient satisfaction was high (8/10) + patients produced physiologic changes in desired direction.	No adherence data
								• Total cost of the system about $9000	

* = 8 weeks (56 days) to 11.4 weeks (80 days) (=recommended duration of treatment. With 8 lessons, each lesson advised to be completed in 7–10 days) Actual average treatment duration = 3.6 months (suggesting it took ~2wks per lesson).** = First group of adolescents/ caregivers for interviews and concept mapping: 12 (ages 12–17, adolescents), 9 (ages 30–55, caregivers) 12 (adults, clinicians).*The same procedure was used to recruit a second group of adolescents and their caregivers and clinicians to evaluate the prototype website: ?12? (ages 12–17, adolescents), ?9? (34–55, “mothers”) and ?12? (clinicians, adults). *** = 44 (ages 25–63) for ODA group feasibility and utility study aim, 31 (ages 25–59) in ODA+ BT group, 31 (ages 26–58) in ODA- group (matched controls). **** = *N* = not specified (“a number of patients”) A description of 2 patients with headache who received biofeedback with the ProComp remote system was provided, however they indicate there are more patients who have received this treatment and state a study to objectively evaluate equivalency between telehealth and in-vivo treatment is underway. HI, Headache Index; Tx, Treatment; HA, Headache; HA days, Days with headache; CPC, Chronic pain coping; DAS, Depression anxiety stress; QoL, Quality of life; LoC, Locus of control

As indicated in Table 2, the outcomes measured from the various studies ranged considerably. About one third of studies (7/23, 30 %) used measures of headache occurrence as outcomes such as a 50 % reduction in headache frequency, headache intensity or headache index as an outcomes measure. The most frequent outcome assessed was acceptability of/satisfaction with the intervention, with 9/23 (39 %) studies assessing this. Five studies (22 %) used medications or a medication index for assessment. 6/23 (26 %) studies used measures of depression, anxiety and/or stress. Only 2/23 (9 %) studies evaluated cost. Three (13 %) used self-efficacy and/or locus of control.

**Table 2:**
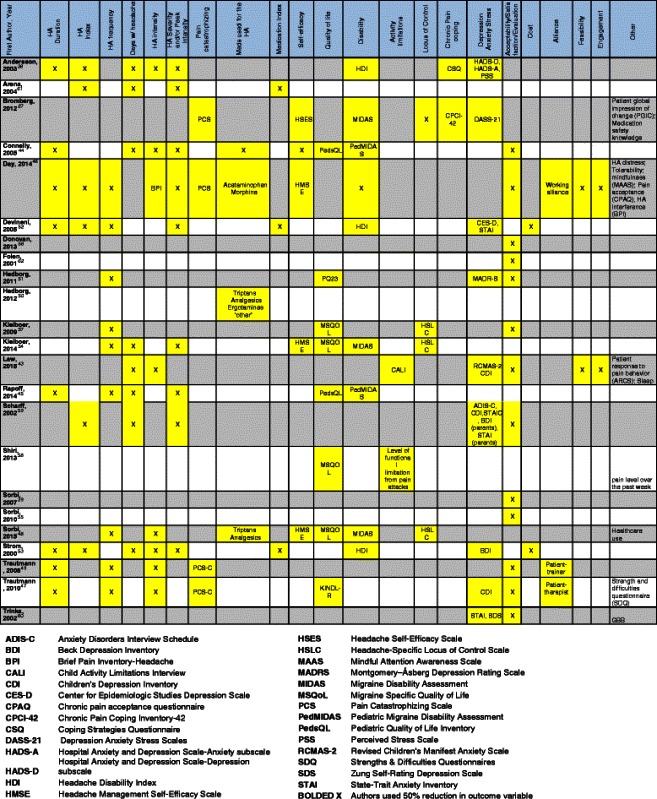
Outcomes table ([27, 38–57] 58–62)

Many of the studies were considered “pilot” studies and thus had a small number of subjects, limiting the ability to detect differences between treatment groups and controls. 16/23 (70 %) studies had a *N* ≥ 30. Of the 16, one study by Bromberg et al. [27] had technical issues leading to the loss of data. Of the remaining 15 studies, six had statistically significant positive findings for their respective primary outcomes (frequency and/or intensity and/or disability and/or medications taken) in the electronic behavioral intervention group compared to controls. No studies addressed power analysis.

Looking at the 9/23 (39 %) studies that offered CBT interventions with *N* ≥ 30, there was a positive outcome in headache frequency, severity, intensity and/or migraine medications taken in 4/9 (44 %) studies. Of the 3/23 (13 %) studies which used biofeedback as part of the behavioral intervention, only two (67 %) had *N* ≥ 30 and one (33 %) of these studies had a decrease in peak intensity. Of the 8/23 (35 %) studies using relaxation, 7 (88 %) had *N* ≥ 30 and of these, only 2/7 (29 %) had positive outcomes (frequency and peak intensity).

One study by Bromberg and colleagues evaluated whether there was a dose response by test determining whether more time spent on the intervention website resulted in a greater treatment effect. The researcher divided the treatment group into low-dose and high-dose groups. Participants who used the site the most reported greater increases in feelings of self-efficacy from baseline to post-intervention (*t* = 5.41, *P* < .0010), 3 months (*t* = 4.53, *P* < .0010) and 6 months (*t* = 4.64, *P* < .0010) but outcome variables aside from self-efficacy were not affected [27]. This study also performed a completer analysis (i.e. whether there was a different intervention effect for participants who completed all study assessments compared with those who did not). For all but 2 outcomes (self-efficacy and headache locus of control), no difference in treatment effect was noted for completers compared with non-completers.

### Discussion

Of the 23 papers on digitally delivered behavioral interventions for headache, all were published after the year 2000 and most (55 %) were published after the year 2010. Overall results suggest moderate to high rates of acceptability and feasibility of using technology to treat headaches, although efficacy data are more limited. Patients were comfortable using a variety of delivery modalities including CD-ROMs in two studies, the internet in 14 studies, personal digital assistants in two studies, custom biofeedback devices in three studies, and a personal audio player in one study. Although the pilot nature of many of these studies makes it difficult to directly compare outcomes, results of these 23 studies can help guide future efforts to develop more effective, engaging, and evidence-based digital interventions for headache.

Focusing on outcomes, results of the 23 studies suggest that patients are willing to use digitally-delivered behavioral headache interventions. The studies used varied headache related outcome measures: headache index, headache duration, headache frequency, headache intensity, medications, self-efficacy, quality of life, sleep, anxiety, depression, distress, and disability. Also, the studies used many diverse outcome measures regarding the use of the technology being studied: satisfaction, feasibility, tolerability, adherence, and engagement among others. The variability in reporting instruments used to record headache symptoms limits direct comparison between studies. However, in an effort to broadly understand the overall impact on headache, we considered which interventions led to a 50 % or greater reduction in headache symptoms seen only in the intervention and not in the control group. Only a minority of studies were able to demonstrate this statistically significant reduction in symptoms. The combination of internet based interventions with CBT had the most positive effect on headache reduction. Given the widespread acceptance of the effectiveness of CBT for headache symptoms and the increasing access as well as comfort that many have with the internet, the successful combination is not surprising. Internet-delivered CBT has also been an area of intense research in other fields such as psychiatry in exploring applications for treating depression [28–30] and in pain medicine for reducing discomfort. [31–33] Along these lines, given the high prevalence of psychiatric comorbidity and migraine [34], future studies can be done to explore whether these electronic behavioral interventions help pain outcomes and the psychiatric symptoms.

The results of many of the reviewed studies must be understood in terms of several common themes. Drop-out rates for the digital interventions ranged widely. Adherence is a common concern for headache medication [35, 36], with estimates that anywhere from 25 % to 94 % of adults adhere to headache treatment regimens [35]. Thus, adherence is also a concern for digital interventions. Recent studies of computer based CBT delivered for purposes outside of headache have suggested that the average user may only complete a single session of computer based CBT [37] and our review suggests the same issue may occur when using computer CBT for headache. These adherence rates in the review may actually be higher than in clinical practice as some studies tied compensation to adherence and others offered personal check-ins or other study staff interventions to encourage adherence. Given the chronic nature of many headache conditions, long term adherence or adherence with booster sessions may be important if digital interventions are to have long term efficacy. The average duration of studies was only 6 weeks, rendering it difficult to fully understand how engaged patients will be with digital tools that may be used over a period of months instead of weeks. Future studies can be done to better assess adherence. Future studies can evaluate the need for booster sessions to help maintain skills. Engagement of patients, families, and treating clinicians in the development, refinement, and post study iterative improvement in the intervention would likely increase adherence and potency of the treatment. Studies can also be done to determine whether the psychiatric comorbidities such as anxiety and depression play a role in adherence with electronic behavioral treatments for headache. In addition, whether these evidence-based treatments can effectively be delivered electronically outside of the clinical setting remains to be seen.

Beyond concerns regarding adherence, understanding the impact of these digitally delivered behavioral interventions for headache is difficult because of the numerous confounding variables in many of the individual studies. But rather than being just a cause for caution, these confounding variables also suggest important research agenda items in seeking to understand the mechanism and efficacy of these interventions. For example, numerous studies involved human contact, be it in the form of a research assistant checking in to a therapist offering personalized feedback, although the impact of such additional human interaction and feedback was hardly explored. In our review, we found one study conducted to explicitly determine the role of such human contact; the study by Andersson and colleagues examined whether telephone contact by a therapist improved adherence and headache related disability, stress, and coping outcomes when used with an internet-based cognitive behavioral self-help program. However, therapist-initiated telephone calls did not influence the results [38]. Also, many of the interventions employed technologies that prompted use between once a day to several times per day but again the impact and optimal number of prompts was examined in only one study, in which the investigators noted that users displayed some annoyance about the number of digital prompts they received [39]. Along similar lines, no studies looked at the right “dose” of behavioral interventions, e.g., how many CBT sessions are necessary, the most effective length of each CBT session, and if and when should there be refresher lessons. Of the reviewed papers, the average number of sessions or lessons was six and was static and fixed for each study. Finally, few studies employed blinded controls (e.g., sham CBT) making it hard to understand the true placebo effect of receiving what may be perceived as ‘high tech treatment.’ However, per the Behavioral Clinical Headache Trials Guidelines, the placebo is not a realistic expectation of behavioral trials anyway [40].

In this era of increasing interest in mobile health, smartphone apps, and wearables, it was interesting to note the paucity of literature exploring the mobile delivery of behavioral interventions for headache. This is especially interesting as smartphones can fulfill many of the delivery modalities explored in the literature (cd-rom, internet, personal digital assistants, audio) and also offer all the interventions offered in the literature (CBT, self-management, relaxation, and biofeedback). With ownership of smartphones in the general population at 64 % in 2015 [41] and expected to increase in 2016, it seems likely that smartphones will may offer an accessible and practical tool for behavioral interventions for treatment of headache. However, further data is needed on those who do not have access or ability to use a smartphone in order to understand the true potential. Still, the lack of any outcomes literature on this topic reflects a critical opportunity of researchers.

Despite the paucity of data on smartphone interventions, a quick search of the Apple or Android app marketplaces shows that this lack of a clinical evidence base has not stopped industry or consumer interest in headache intervention apps. Recent reviews have called into question the quality of these headache apps on the commercial marketplaces [42]. Assuming that the same complexities of efficacy and adherence that were noted in the 14 studies of internet delivered CBT for headache may also be applicable for smartphone delivered interventions—such concerns about the utility of commercially based headache apps seem valid although more research is needed here.

Several limitations must be considered in light of our methodology. As a literature review, we only report on published clinical studies and ignore industry and private efforts to use technology to deliver behavioral interventions for headache. While it is likely that industry efforts have achieved results beyond those noted in our review—validating such technologies in clinical studies remains critical. Also, we did not evaluate the quality of CBT, biofeedback or PMR delivered in the reviewed papers and it is possible that differences in the delivery of the behavioral intervention itself may have impacted outcomes. For example, one study used not individual but family based CBT [43]. In addition, we did not control for study methodology with some studies offering subjects more encouragement and assistance which may have also impacted adherence rates. Quantifying such support is difficult as many studies were vague or did not report on exact amount or quantity of assistance provided in using the technology. Finally, we acknowledge that there are no established data on the effect sizes of the electronic behavioral interventions for headache. We used *N* ≥ 30 to collectively describe studies that were not just very small pilot studies.

## Conclusion

Through this systematic review we have explored the literature on digitally delivered behavioral interventions for headache. While results suggest feasibility, data on efficacy and adherence is harder to interpret. The lack of studies on smartphones is notable and presents an important research opportunity going forward.

## Appendix

### Search strategy

PUBMED, Embase and Psycinfo, 2000-December 11, 2015, restricted to English

The search strategy was as follows:

(“Headache”[Mesh] OR “Headache Disorders”[Mesh] OR “Headache Disorders, Primary”[MeSH] OR “Migraine Disorders”[MeSH] OR “Tension-Type Headache”[Mesh] OR headache[tiab] OR headaches[tiab] OR migraine[tiab] OR migraines[tiab] OR migranous[tiab] OR “head pain”[tiab] OR “head pains”[tiab] OR Cephalalgia[tiab] OR Cephalalgias[tiab] OR “Cranial Pains”[tiab] OR “Cranial Pain”[tiab] OR “tension-type headache”[tiab] OR “tension-type headaches”[tiab] OR “tension headache”[tiab] OR “tension headaches”[tiab] OR headache[ot] OR headaches[ot] OR migraine[ot] OR migraines[ot] OR “head pain”[ot] OR Cephalalgia[ot] OR Cephalalgias[ot] OR “Cranial Pain”[ot] OR “tension-type headache”[ot] OR “tension-type headaches”[ot] OR “tension headache”[ot]) AND (“Mobile Applications”[Mesh] OR “Internet”[Mesh] OR “Telemedicine”[Mesh] OR telemedicine[tiab] OR “Telephone”[Mesh] OR telephone[tiab] OR “Computers”[Mesh] OR “audiovisual aids”[Mesh] OR “mp3-player”[Mesh] OR “Therapy, Computer-Assisted”[Mesh] “electronics, medical”[Mesh] OR “Electronics”[Mesh] OR “software”[MeSH] OR “online systems”[MeSH] OR “cell phones”[MeSH] OR smartphone[tiab] OR “smart phone”[tiab] OR iphone[tiab] OR mobile[tiab] OR cellphone[tiab] OR cellphones[tiab] OR “cell phone”[tiab] OR “cell phones”[tiab] OR internet[tiab] OR mhealth[tiab] OR “mobile health”[tiab] OR telehealth[tiab] OR eHealth[tiab] OR computer[tiab] OR computers[tiab] OR electronic[tiab] OR electronics[tiab] OR “medical electronics”[tiab] OR “world wide web”[tiab] OR “web based”[tiab] OR device[tiab] OR devices[tiab] OR technology[tiab] OR technologies[tiab] OR tech[tiab] OR “remote delivery”[tiab] OR “remotely delivered”[tiab] OR “mp3 player”[tiab] OR ipod[tiab] OR iPad[tiab] OR online[tiab] OR “communication technology”[tiab] OR wireless[tiab] OR “medical informatics”[Mesh] OR digital[tiab] OR online[ot] OR smartphone[ot] OR “smart phone”[ot] OR iphone[ot] OR mobile[ot] OR cellphone[ot] OR cellphones[ot] OR “cell phone”[ot] OR “cell phones”[ot] OR internet[ot] OR mhealth[ot] OR “mobile health”[ot] OR telehealth[ot] OR eHealth[ot] OR computer[ot] OR computers[ot] OR electronic[ot] OR electronics[ot] OR “medical electronics”[ot] OR “world wide web”[ot] OR “web-based”[ot] OR device[ot] OR devices[ot] OR technology[ot] OR technologies[ot] OR tech[ot] OR “mp3 player”[ot] OR ipod[ot] OR iPad[ot] OR online[ot] OR wireless[ot] OR telephone[ot] OR “telephone administered”[tiab]) AND (“Behavior Therapy”[Mesh] OR “Biofeedback, Psychology”[Mesh] OR “Desensitization, Psychologic”[Mesh] OR “relaxation therapy”[Mesh] OR “neurofeedback”[Mesh] OR “Mind-Body Therapies”[Mesh] OR “Cognitive Therapy”[Mesh] OR “meditation”[Mesh] OR “muscle relaxation”[Mesh] OR “self care”[MeSH] OR “behavior therapy”[tiab] OR “behaviour therapy”[tiab] OR “behavior therapies”[tiab] OR “behaviour therapies” OR “behavioral therapies”[tiab] OR “behavioural therapies”[tiab] OR “behavioral treatment”[tiab] OR “behavioural treatment”[tiab] OR “behavioral treatments”[tiab] OR “behavior treatment”[tiab] OR “behavioral approach”[tiab] OR “behavioral approaches”[tiab] OR biofeedback[tiab] OR biofeedbacks[tiab] OR neurofeedback[tiab] OR “cognitive therapy”[tiab] OR “cognitive therapies”[tiab] OR “cognitive behavioral therapy”[tiab] OR “cognitive behavioral treatment”[tiab] OR “cognitive behavioral treatments” OR “cognitive behavioral therapies”[tiab] OR “Cognitive-behavioral interventions”[tiab] OR “Cognitive-behavioral intervention”[tiab] OR “Psychologic Desensitization”[tiab] OR “relaxation techniques”[tiab] OR “relaxation technique”[tiab] OR “relaxation therapy”[tiab] OR “relaxation therapies”[tiab] OR “guided relaxation”[tiab] OR meditation[tiab] OR meditate[tiab] OR “Progressive muscle relaxation”[tiab] OR “neurofeedback”[tiab] OR “guided imagery”[tiab] OR “music therapy”[tiab] OR “mind-body therapy”[tiab] OR “mind-body therapies”[tiab] OR behavioral training [tiab] OR self-management [tiab] OR self-care [ot] OR psychological therapy [tiab] OR psychological therapies [tiab] OR “psychosocial support”[tiab] OR “behavior therapy”[ot] OR “behaviour therapy”[ot] OR “behavior therapies”[ot] OR “behaviour therapies” OR “behavioral therapies”[ot] OR “behavioural therapies”[ot] OR “behavioral treatment”[ot] OR “behavioural treatment”[ot] OR “behavioral treatments”[ot] OR “behavior treatment”[ot] OR “behavioral approach”[ot] OR “behavioral approaches”[ot] OR biofeedback[ot] OR neurofeedback[ot] OR “cognitive therapy”[ot] OR “cognitive therapies”[ot] OR “cognitive behavioral therapy”[ot] OR “cognitive behavioral treatment”[ot] OR “cognitive behavioral treatments” OR “cognitive behavioral therapies”[ot] OR “Cognitive-behavioral interventions”[ot] OR “Cognitive-behavioral intervention”[ot] OR “relaxation techniques”[ot] OR “relaxation technique”[ot] OR “relaxation therapy”[ot] OR “guided relaxation”[ot] OR meditation[ot] OR meditate[ot] OR “Progressive muscle relaxation”[ot] OR “neurofeedback”[ot] OR “guided imagery”[ot] OR “music therapy”[ot] OR “mind-body therapy”[ot] OR “mind-body therapies”[ot] OR behavioral training [ot] OR self-management [ot] OR self-care[ot] OR psychological therapy[ot] OR psychological therapies[ot])

**Table 3:** Various descriptions of cognitive behavioral therapy (CBT) conducted in the studies

Connelly, 2005[44]	A CD-ROM program was provided to give additional strategies to help manage head pain.
Rapoff, 2014 [45]	“…lessons on how to use various empirically supported cognitive-behavioral treatments to self-manage recurrent headaches…focused on problem-solving and stress management, and targeted pain behavior and parental response to pain.”
Day, 2014 [46]	“8-week MBCT for depression Protocol” Protocol: Segal Z, Williams JM, Teasdale J. Mindfulness-based Cognitive Therapy for Depression: A New Approach to Preventing Relapse. New York: The Guilford Press; 2002.
Bromberg, 2012 [27]	“Lessons—interactive instruction for learning practical pain self-management skills and strategies, and how to apply them to solve problems. Tools—visual and graphic interactive learning experiences that allow users to actively manipulate information to construct knowledge and learn to solve problems. Self-assessments—structured sets of questions that help users to reflect on and learn what skills and knowledge they have, where their relative strengths and deficits are, and how to identify what behaviors to target for change.”
Law, 2015 [43]	“Psychological therapy included face-to-face cognitive behavioral therapy for pain management and/or biofeedback. In addition, participants received access to an Internet CBT program (Web-based Management of Adolescent Pain; Web-MAP). The design and treatment content of Web-MAP was identical to the original version of the program.” Original program: Palermo TM, Wilson AC, Peters M, Lewandowski A, Somhegyi H. Randomized controlled trial of an internet-delivered family cognitive-behavioral therapy intervention for children and adolescents with chronic pain. Pain. 2009;146:205-213.
Trautmann, 2010 [47]	“Adapted from the manualized face-to-face group therapy program devised by Denecke and Kro¨ ner-Herwig for children with recurrent headache.” Manual in German: Denecke, H., & Kroner-Herwig, B. (2000). Kopfschmerztherapie mit Kindern und Jugendlichen. Ein Trainingsprogramm.Gottingen: Hogrefe. “The first module presented education on headaches, the second unit focused on stress management (perception of own stress symptoms, coping with stress). In the following modules the participants acquired various skills including “cognitive restructuring (identification of dysfunctional cognitions regarding headache and stress and identifying functional cognitions).”
Sorbi, 2015 [48]	Focused on “graded tasks, specific goal setting and review of behavioral goals; behavior self-monitoring with teaching the use of prompts and cues, feedback on performance and barrier identification; behavioral modeling and social comparison, a focus on time- or stress-management, and relapse prevention. Adapted from several studies and later on turned into a module: Sorbi MJ and Swaen SJ. Protocollaire behandeling van patienten met migraine en spanningshoofdpijn:Ontspannings training en cognitieve training (Training according to protocol in migraine and tension-type headache: Relaxation training and cognitive training). In: Keijsers GPJ, van Minnen A and Hoogduin CAL(eds) Protocollaire behandelingen in de ambulante geeste-lijke gezondheidszorg (Treatment according to protocol in ambulatory mental health care). Houten: Bohn Stafleuvan Loghum, 2004, pp.219–260.
Trautmann, 2008 [49]	6 self-help sessions (focusing on education on headaches, stress management, relaxation, cognitive restructuring, self-assurance strategies, problem solving) based on a face-to-face training manual. Manual: Kroner-Herwig, B. and Denecke, H. (2002). Cognitive-behavioural therapy of paediatric headache. Are there any differences in efficacy between a therapist-administered group training and a self-help format? Journal of Psychosomatic Research, 53, 1107–1114.
Hedborg, 2012 [50, 51]	53-page training program aimed at improving stress coping skills and divided into the following topics: stress physiology, physical activity, diet, thought patterns, handling of emotions, and attitudes. Training Program: Hedborg K and Muhr C. Multimodal behavioral treatment of migraine: an Internet-administered, randomized, controlled trial. Ups J Med Sci 2011; 116: 169–186.
Andersson, 2003 [38]	“Cognitive-behavioral techniques for handling negative thoughts and core beliefs were included.”
